# Supplementary material for: Synergistic application of artificial intelligence and response surface methodology for predicting and enhancing in vitro tuber production of potato (Solanum tuberosum)
Source: PLoS One. 2025 Jun 24;20(6):e0325754. doi: 10.1371/journal.pone.0325754 (PMC12186905; doi:10.1371/journal.pone.0325754)
Supplement: Attachment — Submitted filename: Reviwes.docx [file pone.0325754.s001.docx]

1. The paper is flows well. The necessity of integration of Machine Learning model is well explained.

2. They have executed all the experiments to evaluate the performance of the machine learning models and it is showing in the manuscript. All the experimental results are already represented in the table. Just refer the table no requirements of writing all the values in the text.

3. The section is not represented properly. Write at least one line between section 3 and subsection 3.1.

4. A literature review section may include in the manuscript.
